# A comparison of cemented femoral fixation via anterior versus posterior approach total hip arthroplasty: an analysis of 60,739 total hip arthroplasties

**DOI:** 10.1177/11207000241239914

**Published:** 2024-03-26

**Authors:** Wayne Hoskins, Sophie Corfield, Yi Peng, Stephen E Graves, Roger Bingham

**Affiliations:** 1 Faculty of Medicine, Dentistry and Health Sciences, The University of Melbourne, Parkville, VIC, Australia; 2 Traumaplasty. Melbourne, East Melbourne, VIC, Australia; 3 Australian Orthopaedic Association National Joint Replacement Registry, Adelaide, South Australia, Australia

**Keywords:** Osteoarthritis, prosthesis surgical approach, total hip replacement

## Abstract

**Background:**

Anterior approach total hip arthroplasty (THA) decreases the rate of dislocation but increases femoral-sided complications in the way of periprosthetic fractures and component loosening. A cemented prosthesis may reduce femoral-sided complications and improve the risk:benefit profile of anterior approach THA.

**Methods:**

Data from the Australian National Joint Replacement Registry were analysed for patients undergoing primary THA via the anterior or posterior approach using a cemented polished femoral stem from January 2015 to December 2021. The primary outcome measure was the cumulative percent revision (CPR) for all causes and CPR for femoral component loosening and fracture. The CPR for the primary outcome measures were compared between the anterior and posterior approach and adjusted for age, sex, ASA score, BMI and femoral head size.

**Results:**

The study included 60,739 THAs with cemented stems (10,742 anterior, 49,997 posterior). The rate of revision of the anterior versus the posterior approach did not significantly differ (HR 0.87 (95% CI, 0.74–1.03), *p* = 0.100). Anterior approach THA had a significantly higher rate of revision for femoral component loosening (HR 5.06 [95% CI, 3.08–8.30], *p* < 0.001); and a decreased rate of revision for infection (HR 0.59 [95% CI, 0.43–0.81], *p* = 0.001) and dislocation/instability (HR 0–3 months 0.48 [95% CI, 0.27–0.83], *p* = 0.008; HR >3 months 0.30 [95% CI, 0.15–0.61], *p* < 0.001). There was no difference in the rate of revision surgery for fracture between the 2 approaches (HR 1.01 [95% CI, 0.71–1.43]), *p* = 0.975).

**Conclusions:**

There is no significant difference in overall revision rates with cemented femoral fixation performed with an anterior or posterior approach. Cemented fixation performed with the anterior approach partly mitigates femoral complications with no difference in the revision rate for fracture but an increased rate of femoral component loosening.

## Background

When compared with the posterior approach, the anterior approach for total hip arthroplasty (THA) has a reduced rate of revision surgery for dislocation,^1^ but an increased rate of periprosthetic femur fracture and component loosening,^2–7^ producing equivalent overall revision rates.^4^ The increase in femoral-sided complications is the result of a more difficult exposure to the femur, producing technical challenges with achieving a correct component entry point, broaching and implant insertion. This can result in under-sizing and varus malposition of the femoral component.^8–10^ Femoral complications may be magnified by cementless fixation, and certain stem types,^11,12^ due to the requirement for an immediate press fit that is axially and rotationally stable and correctly sized.

Compared with cementless fixation, cemented femoral fixation is known to have fewer revisions for periprosthetic fracture,^13^ and fewer revisions for component loosening, particularly in patients with advancing age.^14,15^ As such, cemented fixation may be an option in anterior approach THA to reduce femoral-sided complications, whilst preserving the inherent advantages of improved stability with the approach. However, the difficulties with femoral exposure, preparation and stem insertion may persist despite the use of cement and impact femoral prosthesis survivorship. It is unknown whether the anterior approach may impact arthroplasty survivorship through potential comprises to cementing technique, the cement mantle integrity, or femoral stem position on insertion, with the quality of the cement mantle most highly related to prosthesis longevity and the process of component loosening.^16–20^

The aim of this study was to compare the overall component survivorship, femoral-sided revisions, and the different reasons for revision of cemented femoral prostheses performed using the anterior and posterior approach, using data from a large national joint replacement registry. The study population included all patients with a primary diagnosis of osteoarthritis (excluding all other diagnoses) who underwent a THA procedure via the anterior or posterior approach using a cemented smooth polished femoral stem.

## Materials and methods

The Australian Orthopaedic Association National Joint Replacement Registry (AOANJRR) includes information on almost 100% of arthroplasty procedures performed in Australia and it commenced data collection for surgical approaches for THA in 2015. Registry data are validated against patient-level data provided by each of the State and Territory Health Departments with the use of a sequential, multi-level matching process. A matching program is run monthly to search for all primary and revision arthroplasty procedures recorded in the Registry that involved the same side and joint for the same patient, thus enabling each revision to be linked to the primary procedure. Data is matched by the Australian Institute of Health and Welfare’s National Death Index to obtain information on the date of death. The Registry records the reasons for revision and the type of revision THA.

The study included procedures reported to the AOANJRR from 01 January 2015 – when the type of surgical approach was first recorded by the AOANJRR – to 31 December 2021. The study population included all patients with a primary diagnosis of osteoarthritis (excluding all other diagnoses) who underwent a THA procedure via the anterior or posterior approach using a cemented smooth polished femoral stem. The anterior approach is defined as direct anterior (Judet, Smith-Peterson, Heuter intervals) and not anterolateral (Watson-Jones interval, Hardinge) approach. The exclusion criteria were diagnoses other than osteoarthritis, all procedures using non-cross-linked polyethylene (non-XLPE) or metal/metal bearings and cemented femoral stems with a matt finish due to their higher rates of revision.^21^ All cemented smooth polished femoral stems were grouped together because there is little variation in the surgical technique or the outcome of surgery.^21^

The primary outcome measures were the cumulative percent revision (CPR) for all causes and the CPR for a diagnosis of femoral stem loosening and fracture. The secondary outcome measure was the CPR for other causes of revision. The effect of age, sex, American Society of Anesthesiologists (ASA) score, body mass index (BMI), and femoral head size were also considered because of the potential for confounding. The different causes of revision and different revision types were compared.

### Statistical analysis

Time to first revision was described using Kaplan-Meier estimates of survivorship, with right censoring for death or closure of the database at the time of analysis. The unadjusted CPR was estimated as the complement in probability of the Kaplan-Meier survivorship estimate, with 95% confidence intervals (CI) estimated using pointwise Greenwood estimates for the standard error. Hazard ratios (HR) from Cox proportional hazard models, adjusting for age, sex, ASA score, BMI, and femoral head size were used to compare the rate of revision between groups. Regression analyses were restricted to procedures with complete data for all covariates. The assumption of proportional hazards was checked analytically for each model. If the interaction between the predictor and the log of time was statistically significant in the standard Cox model, then a time-varying model was estimated. Time points were iteratively chosen until the assumption of proportionality was met and the hazard ratios were calculated for each selected time period. In the results, if no time period was specified then the hazard ratio was proportional over the entire follow-up period. All tests were two-tailed at the 5% level of significance. Analysis was performed using SAS version 9.4 (SAS Institute Inc., Cary, NC, USA).

## Results

The study included 60,739 THAs with cemented polished femoral stems (10,742 anterior and 49,997 posterior). The mean age of patients receiving anterior approach THA was similar to the posterior approach (73.9 ± 9.0 years vs. 71.5 ± 10.3 years), as was BMI (27.9 ± 5.8 vs. 29.5 ± 6.6 kg/m^2^), and ASA, although more females received the anterior approach (67.1% vs. 60.4%) and the distribution of age and BMI was different (Table 1). The 6-year CPR for the anterior approach was 2.7% (95% CI, 2.1–3.3) and for the posterior approach 2.8% (95% CI, 2.6–3.0). There were *n* = 167 (1.6%) revisions with the anterior approach and *n* = 1043 (2.1%) for the posterior approach, with the most common causes of revision being infection (*n* = 43 (0.4%) vs. n = 405 (0.8%), dislocation/instability (*n* = 24 (0.2%) vs. *n* = 299 (0.6%), fracture (*n* = 40 (0.4% vs. *n* = 204 (0.4%) and femoral component loosening (n = 31 (0.3%) vs. *n* = 37 (0.07%) (Table 2).

**Table 1.** Summary of primary total conventional hip replacement since 2003 (primary diagnosis osteoarthritis).

Variable		Anterior	Posterior
**Follow-up** (years)			
	Mean ± SD	2.6 ± 1.8	3.1 ± 1.9
	Median (IQR)	2.3 (1.1, 3.9)	2.9 (1.4, 4.7)
	Minimum	0	0
	Maximum	7	7
**Age** (years)			
	Mean ± SD	73.9 ± 9	71.5 ± 10.3
	Median (IQR)	75 (69, 80)	73 (65, 79)
**BMI**^a^ (kg/m^2^)			
	Mean ± SD	27.9 ± 5.8	29.5 ± 6.6
	Median (IQR)	27.3 (24.2, 30.9)	28.7 (25.3, 32.8)
**Age group** (years)			
	<55	303 (2.8%)	3,186 (6.4%)
	55–64	1,237 (11.5%)	8,385 (16.8%)
	65–74	3,756 (35%)	17,493 (35%)
	⩾75	5,446 (50.7%)	20,933 (41.9%)
**Gender**			
	Male	3,536 (32.9%)	19,791 (39.6%)
	Female	7,206 (67.1%)	30,206 (60.4%)
**ASA score** ^b^			
	ASA 1	633 (5.9%)	2,611 (5.2%)
	ASA 2	5,565 (51.9%)	25,494 (51%)
	ASA 3	4,353 (40.6%)	20,858 (41.8%)
	ASA 4	177 (1.6%)	976 (2%)
	ASA 5		2 (0%)
**BMI category**^a^ (kg/m^2^)			
	Underweight (<8.50)	146 (1.4%)	483 (1%)
	Normal (18.50–24.99)	3118 (29.8%)	10,545 (21.7%)
	Pre Obese (25.00–29.99)	3983 (38.1%)	17,435 (35.9%)
	Obese Class 1 (30.00–34.99)	2249 (21.5%)	12,053 (24.8%)
	Obese Class 2 (35.00–39.99)	719 (6.9%)	5,400 (11.1%)
	Obese Class 3 (⩾40.00)	246 (2.4%)	2,644 (5.4%)
**Fixation**			
	Cemented	190 (1.8%)	2855 (5.7%)
	Hybrid (femur cemented)	10,552 (98.2%)	47,142 (94.3%)
**Femoral head size**			
	<32mm	566 (5.3%)	5631 (11.3%)
	32mm	4822 (44.9%)	23,291 (46.6%)
	>32mm	5354 (49.8%)	21,075 (42.2%)
**Bearing surface**			
	Ceramic/ceramic	1295 (12.1%)	2884 (5.8%)
	Ceramic/XLPE	4641 (43.2%)	16,007 (32%)
	Metal/XLPE	3989 (37.1%)	29,697 (59.4%)
	Ceramicised metal/XLPE	817 (7.6%)	1409 (2.8%)
**TOTAL**		10,742	49,997

SD, standard deviation; IQR, interquartile range; ASA, American Society of Anesthesiologists; BMI; body mass index.

a 1,718 procedures have unknown BMI.

b 70 procedures have unknown ASA Score.

**Table 2.** Revision diagnosis of cemented polished primary total conventional hip replacement by approach since 2015 (primary diagnosis osteoarthritis).

Revision diagnosis	Anterior			Posterior		
	Number	% Primaries revised	% Revisions	Number	% Primaries revised	% Revisions
Infection	43	0.4	25.7	405	0.8	38.8
Prosthesis dislocation/instability	24	0.2	14.4	299	0.6	28.7
Fracture	40	0.4	24.0	204	0.4	19.6
Loosening, acetabular component revision	11	0.1	6.6	28	0.1	2.7
Loosening, femoral component revision	24	0.2	14.4	26	0.1	2.5
Loosening, other type of revision	1	0.0	0.6	13	0.0	1.2
Loosening, THR (femoral/acetabular) revision	7	0.1	4.2	11	0.0	1.1
Pain	2	0.0	1.2	10	0.0	1.0
Malposition	4	0.0	2.4	9	0.0	0.9
Implant breakage stem	1	0.0	0.6	7	0.0	0.7
Leg-length discrepancy	3	0.0	1.8	5	0.0	0.5
Incorrect sizing	3	0.0	1.8	3	0.0	0.3
Lysis				3	0.0	0.3
Implant breakage acetabular				2	0.0	0.2
Implant breakage acetabular insert	1	0.0	0.6	2	0.0	0.2
Heterotopic bone	1	0.0	0.6			
Other	2	0.0	1.2	16	0.0	1.5
***n* Revision**	**167**	**1.6**	**100.0**	**1043**	**2.1**	**100.0**
***n* Primary**	**10742**			**49997**		

**Figure 1.**
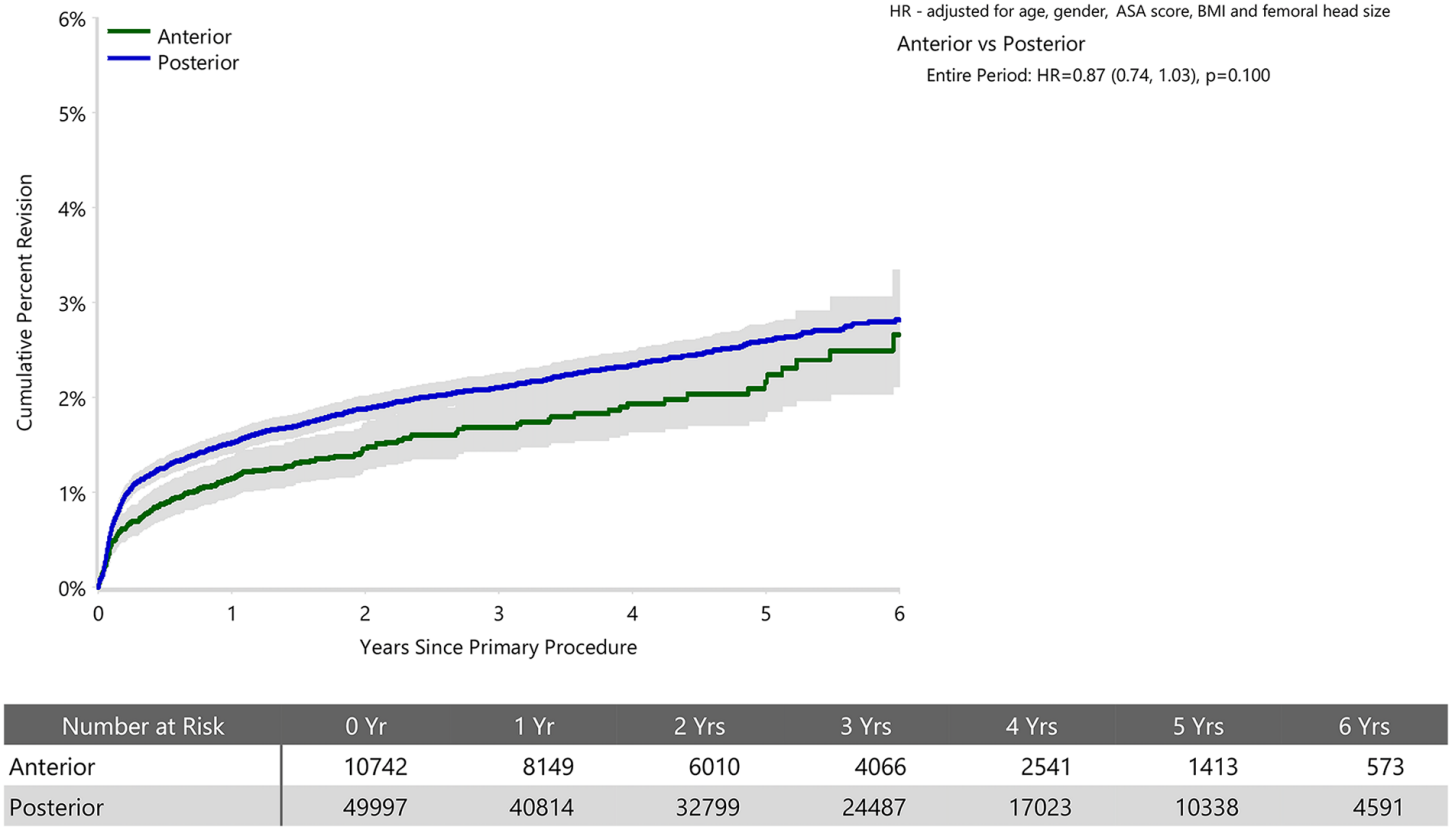
Cumulative percent revision of cemented polished primary total conventional hip replacement by approach since 2015 (primary diagnosis OA).

**Figure 2.**
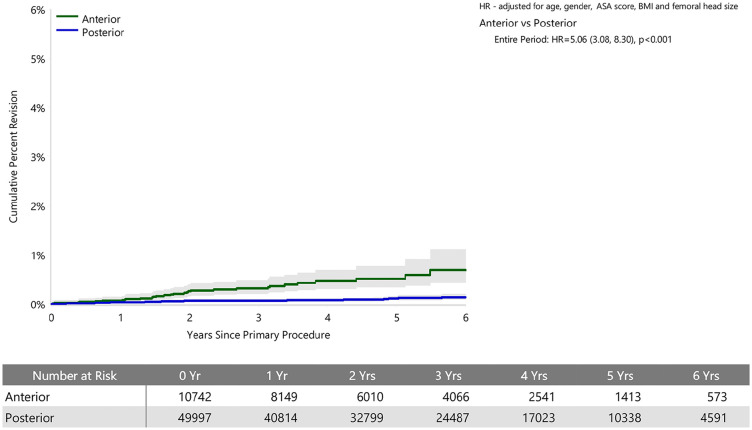
Cumulative percent revision of cemented polished primary total conventional hip replacement by approach since 2015 (primary diagnosis OA, revision for loosening). Femoral Stem Revisions include both THR (Femoral/Acetabular) and Femoral Component revisions.

**Figure 3.**
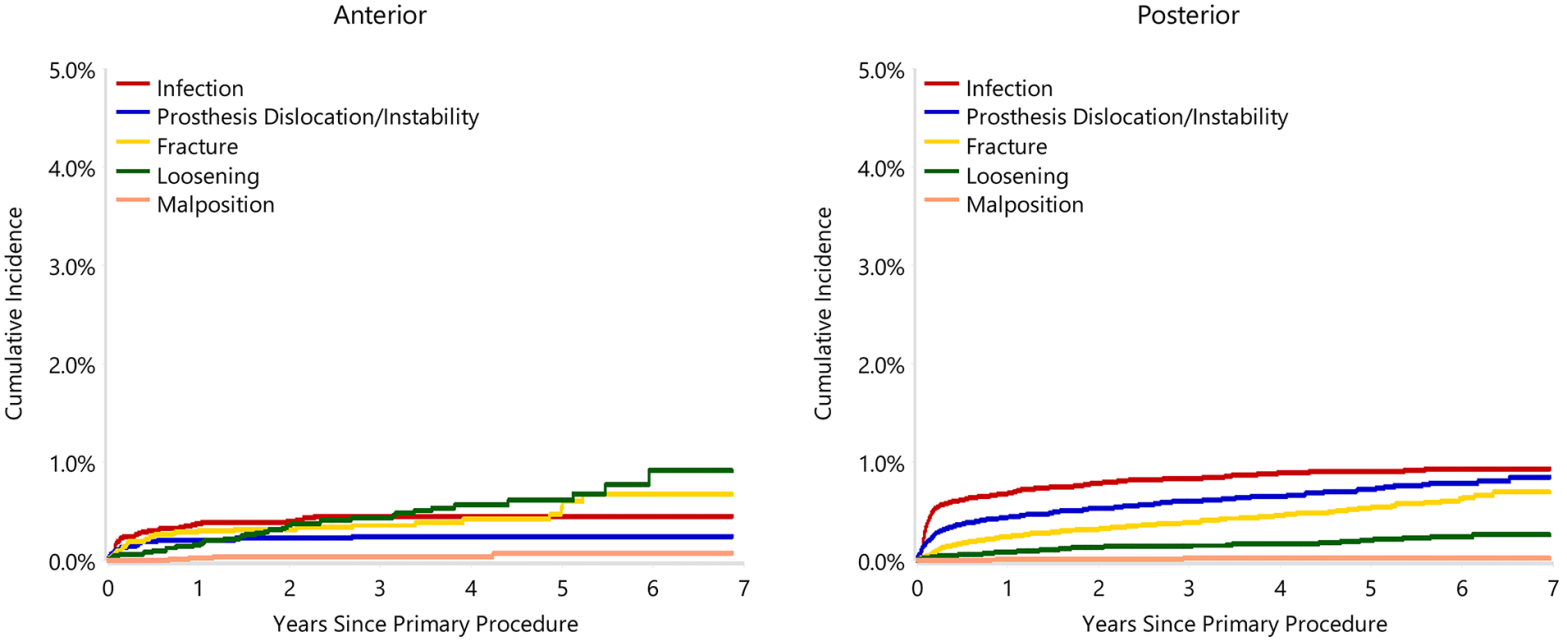
Cumulative incidence revision diagnosis of cemented polished primary total conventional hip replacement by approach since 2015 (primary diagnosis OA).

**Figure 4.**
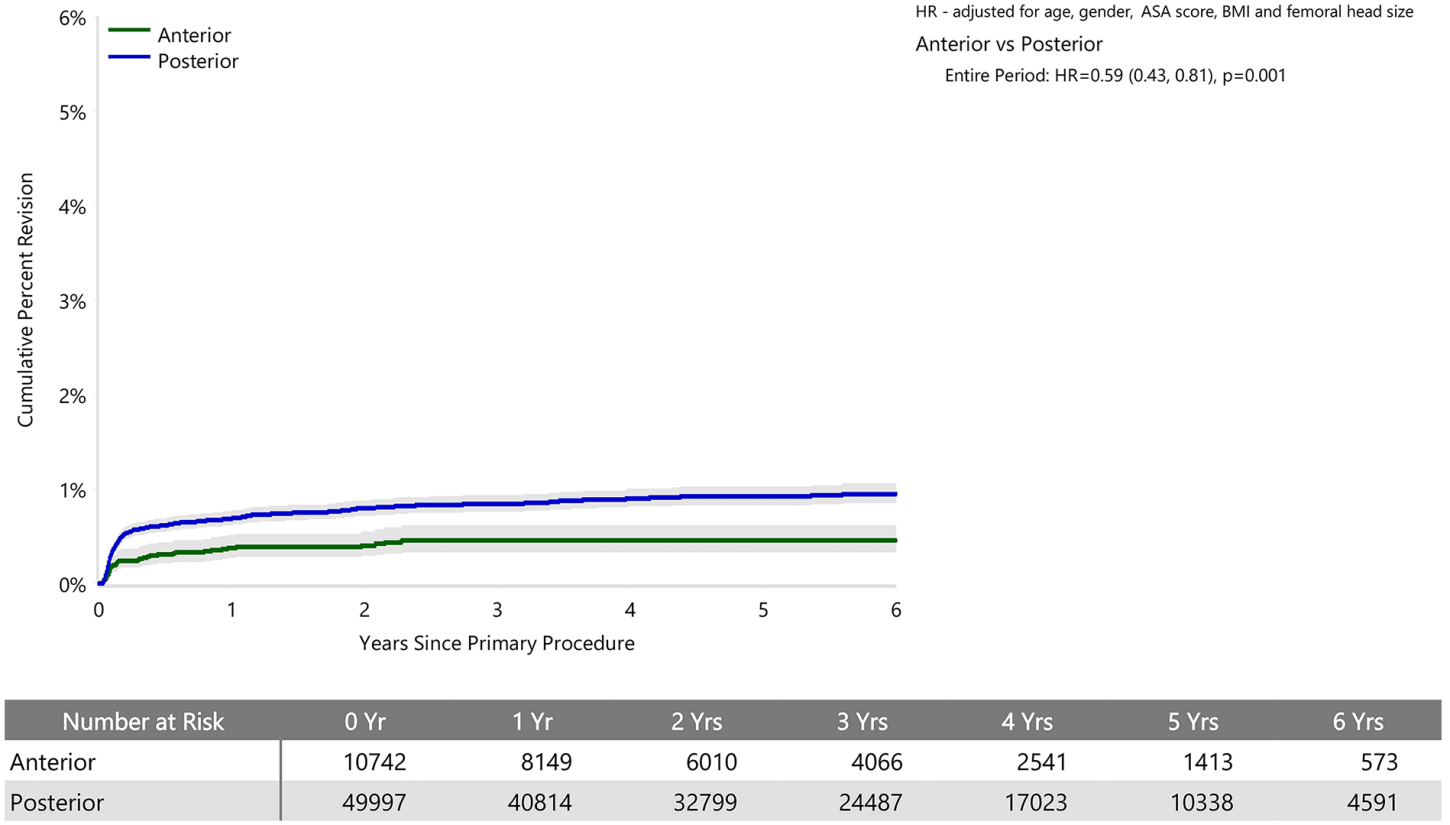
Cumulative percent revision of cemented polished primary total conventional hip replacement since 2015 by surgical approach (primary diagnosis OA, revision for infection).

**Figure 5.**
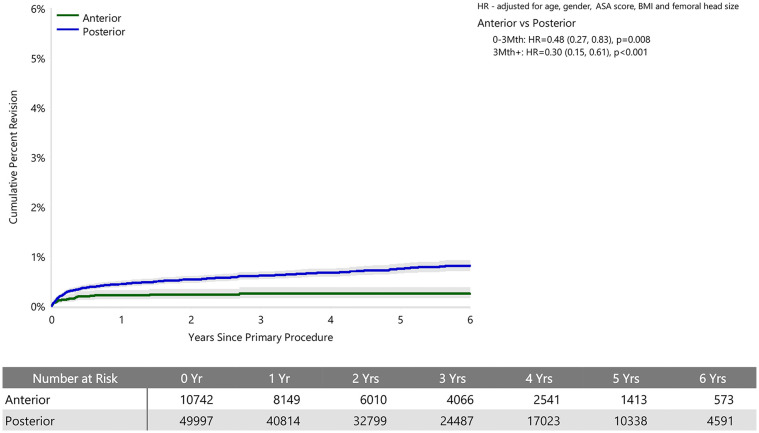
Cumulative percent revision of cemented polished primary total conventional hip replacement since 2015 by surgical approach (primary diagnosis OA, revision for prosthesis dislocation/instability).

When adjusted for age, sex, ASA, BMI, and head size the revision rate of procedures using the anterior approach was not significantly greater than that of procedures using the posterior approach (HR 0.87 (95% CI 0.74, 1.03), *p* = 0.100) (Figure 1). For femoral component loosening, there was a significantly higher rate of revision when the anterior approach was used (HR 5.06 [95% CI 3.08–8.30], *p* < 0.001) (Figure 2). The rate of revision surgery for peri-prosthetic fracture did not significantly differ between the approaches (HR 1.01 [95% CI 0.71–1.43], *p* = 0.975). Overall, the most common reasons for revision with the anterior approach were component loosening, fracture and infection; and for the posterior approach it was infection, dislocation/instability and fracture (Table 2) (Figure 3). The anterior approach had a significantly lower rate of revision for infection (HR 0.59 [95% CI, 0.43–0.81], *p* = 0.001) and dislocation/instability (HR 0–3 months 0.48 [95% CI, 0.27–0.83], *p* = 0.008; HR >3 months 0.30 [95% CI 0.15–0.61], *p* < 0.001) (Figures 4 and 5).

During revision surgery, cemented femoral stems performed using an anterior approach were more likely to require revision of the femoral component and less likely to have only a head/insert exchange (Table 3). The acetabular component was revised in a similar amount between approaches.

**Table 3.** Type of revision of cemented polished primary total conventional hip replacement by approach since 2015 (primary diagnosis osteoarthritis).

Type of revision	Anterior			Posterior		
	Number	% Primaries revised	% Revisions	Number	% Primaries revised	% Revisions
Head/Insert	30	0.3	18.0	414	0.8	39.7
Femoral component	72	0.7	43.1	248	0.5	23.8
Acetabular component	24	0.2	14.4	153	0.3	14.7
THR (femoral/acetabular)	25	0.2	15.0	126	0.3	12.1
Head only	9	0.1	5.4	48	0.1	4.6
Minor components	2	0.0	1.2	16	0.0	1.5
Cement spacer	4	0.0	2.4	15	0.0	1.4
Insert only				8	0.0	0.8
Reinsertion of components				7	0.0	0.7
Removal of prostheses	1	0.0	0.6	7	0.0	0.7
Cement only				1	0.0	0.1
***n* Revision**	**167**	**1.6**	**100.0**	**1043**	**2.1**	**100.0**
***n* Primary**	**10742**			**49997**		

## Discussion

This national registry study did not find evidence of a difference in all-cause revision rates between the anterior and posterior approach in primary THA, where cemented smooth polished femoral stems were used for a median 2.3 and 2.9 years follow-up, respectively, and up to a maximum of 7 years follow-up. However, we did find a difference in the causes and types of revision surgery between the anterior and posterior approach with cemented femoral stem use. Within the follow-up period of this study, there was a statistically significant increase in revisions for femoral component loosening with the anterior approach, no difference for peri-prosthetic fracture and a significant decrease in revisions for infection and dislocation/instability. Given the expected early reliability of fixation provided by cement, femoral component loosening needs to be carefully monitored in anterior approach THA as the incidence may increase further with longer-term follow-up. In this study, over the same time period, femoral loosening was almost non-existent with the posterior approach.

Multiple registries and other studies have found the anterior approach to have a higher incidence of femoral-sided complications.^2–4,6,7,9,22^ Our study showed that there was an increase in femoral component loosening despite using cement fixation, but no difference in peri-prosthetic fracture; a complication often observed with cementless fixation.^4^ It is uncommon to see early aseptic loosening of a cemented femoral stem,^21^ and it was virtually non-existent when THA was performed using the posterior approach in our study. Our results are similar to a comparative study of 360 THA procedures performed with the anterior approach using cemented (*n* = 157) or cementless (*n* = 203) femoral fixation, which showed the cementless group to have a higher rate of femoral complications as a result of peri-prosthetic fracture and component loosening.^23^ Another study of 115 THA or hemiarthroplasty procedures performed using cemented fixation found the lateral alignment of the stem to be significantly more posterior with the anterior approach compared with the posterior approach.^24^ Our results and the available literature suggest that the risks resulting from technical difficulties in adequately exposing and preparing the femur can in part, but not completely, be mitigated with cemented fixation, and cement use would seem a reasonable option for some patients, particularly in the elderly and female patients with poor bone quality; especially with the advantages of lower infection and dislocation rates. It is unclear if cemented femoral component loosening with the anterior approach is due to the approach or due to the surgeon who is not facile with cementing using the anterior approach.

Difficulty with exposure with the anterior approach may produce differences in the cementation technique, the overall cement mantle, and/or femoral component position on insertion. This may jeopardise mechanical fixation or produce cement deficiencies,^25^ contributing to longer-term failure and component loosening.^26,27^ There is limited literature investigating cement fixation performed with the anterior approach. Some studies have shown a higher complication rate using the anterior approach and suboptimal femoral component placement, but the majority of cases have been performed during surgeons’ learning curves.^28–30^ Our national registry data inclusion from 2015 onwards should indicate that many surgeons may be beyond the early learning curve, or well aware of the technical issues and the complications inherent to the anterior approach. Being a registry study, we are unable to fully determine the reasons behind femoral component loosening with the anterior approach. It is presumed that some cases are related to the integrity of the cement mantle. However, if deficiencies in the cement mantle do occur, they should still be a negligible cause of revision within the first decade.^31,32^ This explains our recommendation for longer-term follow-up. A small study found the femoral cement mantle quality achieved through an anterior approach using the Barrack classification systems to be graded A in 39.25% (complete filling without radiolucent lines), B in 53.0% (a radiolucent line covering up to 50% of the cement-bone interface), and C in 7.75% (a radiolucent line covering between 50 and 99% of the cement-bone interface, or incomplete cement mantle) of anteroposterior radiographs and graded A in 40.1%, B in 51.75%, and C 9.5%, with 93% of stems in neutral alignment.^33^ Another study found 92% of stems to be inserted in a neutral position with 85% achieving complete white out of cement in postoperative x-ray.^23^ However, other studies have reported only 71% of stems to be inserted in neutral position using cemented fixation.^24^ There is likely to be variation in the results of individual surgeons which should be further investigated. There is minimal literature comparing the integrity of the cement mantle with an anterior approach to that seen with other THA approaches.^19^ A small study found no significant difference between cemented femoral fixation performed with the anterior and posterior approach regarding the grade or quality of the cement mantle.^24^ A cadaveric study compared the anterior and lateral approaches and found no difference in the quality of the cement mantle, although the stems with the anterior approach had to be inserted in an angulated fashion.^34^ A randomised controlled trial demonstrated minor differences in the cement mantle and implant positioning between the anterior and lateral approaches.^35^ Although we grouped all smooth cemented stems together, it is possible that variations in cemented stem designs, in particular a straight stem, may also influence the cement mantle.^34^ There are also newer philosophies and techniques for cementing in modern practice, including a reduced cement mantle through a line-to-line technique – the so called ‘French Paradox’.^36,37^ Without radiographic assessment of cases in a registry study, or with documentation of the cementing techniques in the AOANJRR, we are unable to comment on the influence this technique may have, and targeted research with radiographic assessment is required to assess this.

There are limitations to this study. The principal limitation is the lack of radiological evidence of surgical performance and grading of the cement mantle. Alternative study designs are required to assess this as no national registry records radiological outcomes. Anterior approach THA in Australia is more commonly performed in private hospitals,^38^ which could influence results. A limitation of the AOANJRR is that the surgeon responsible including their level of training is not recorded, so we are unable to determine the association that the surgeon and location of surgery have on outcomes.^39^ Although we adjusted for multiple confounders, we did not account for surgeon volume, experience, or progression on the learning curve, for either approach. However, our inclusion period commenced in 2015 when approaches were first recorded by the AOANJRR and the first known anterior approach for THA in Australia occurred in 2008, so it is possible that many, but not all, surgeons had been practicing the anterior approach for some time prior to the data collection period of this study. As such most surgeons would have decided on what approach to use based on the known risks and benefits and their own complication profiles and completion of the learning curve. We also do not know whether the complication profile is related to the use of cemented stems, or the surgeons performing the anterior approach. Being a registry study, we were not able to assess the degree of case complexity or surgeon decision-making. However, we attempted to minimise this by only including primary THA performed for a diagnosis of osteoarthritis and adjusting for multiple patient confounders. We recognise that there are other confounding variables that influence the results of THA; particularly spinal pathology and previous history of hip surgery. Ideally, this data would be adjusted for, however, no national joint replacement registry collects any data on the hip-spine relationship, and only a minority (and not the AOANJRR) record previous operations.^40^ Only with enhanced data collection for national registries can improved analyses occur. The AOANJRR only records revision procedures, and there are likely non-revision complications that have not been recorded including closed reductions of dislocation, internal fixation of fractures, and debridement procedures for infection without implant exchange. Regarding periprosthetic fractures, the AOANJRR does not include fracture location, fracture types or a fracture classification; these may be different between the approaches and enhanced data collection could be considered in the future to allow for this. We are also limited with our follow-up, longer-term follow-up is required to assess whether complication rates and causes of revision change. Finally, as with all retrospective cohort research, there are inherent limitations to this study design. In particular, causation cannot be determined, only associations attributed. These inherent limitations would be best addressed through well-designed randomised controlled trials.

## Conclusion

We found no significant difference in the overall revision rates between cemented femoral prosthesis fixation in primary THA performed with either an anterior or posterior approach at a maximum 7 years follow-up (median 2.3 and 2.9 years follow-up, respectively). There was an increased rate of femoral component loosening with the anterior approach, no difference in peri-prosthetic fracture and a decreased rate of revision for infection and dislocation/instability. This suggests the challenges of femoral exposure and preparation associated with the anterior approach, and the subsequent risks related to femoral component preparation and insertion, cannot be completely overcome, but can be partially mitigated with a cemented femoral prosthesis, but the inherent stability advantages of the anterior approach remain. A greater indication for anterior approach THA could be made if femoral complications could be reduced given the benefits of improved prosthesis stability. Cemented femoral fixation would seem to reduce peri-prosthetic fractures, and would seemingly benefit treatment of older patients, female patients, and patients with poor bone quality. Longer-term follow-up of cemented femoral prosthesis fixation performed with anterior approach THA is required to observe changes in complications with time, in particular femoral component loosening, which should be minimal during the first decade with modern cementing techniques and XLPE and is the most common cause of revision. Well designed, randomised controlled trials studies are encouraged to better assess the risk-benefit ratio and long-term outcomes, in particular the rate of femoral loosening and whether it is related more to the approach or the surgeon.

## References

[1] Mohan R Paul HY Hansen EN . Evaluating online information regarding the direct anterior approach for total hip arthroplasty. J Arthroplasty 2015; 30: 803–807. 25697892 10.1016/j.arth.2014.12.022

[2] Angerame MR Fehring TK Masonis JL , et al. Early failure of primary total hip arthroplasty: is surgical approach a risk factor? J Arthroplasty 2018; 33: 1780–1785. 29439894 10.1016/j.arth.2018.01.014

[bibr3-11207000241239914] Eto S Hwang K Huddleston JI , et al. The direct anterior approach is associated with early revision total hip arthroplasty. J Arthroplasty 2017; 32: 1001–1005. 27843039 10.1016/j.arth.2016.09.012

[bibr4-11207000241239914] Hoskins W Bingham R Lorimer M , et al. Early rate of revision of total hip arthroplasty related to surgical approach: an analysis of 122,345 primary total hip arthroplasties. J Bone Joint Surg Am 2020; 102: 1874–1882. 32769807 10.2106/JBJS.19.01289

[bibr5-11207000241239914] Janssen L Wijnands KA Janssen D , et al. Do stem design and surgical approach influence early aseptic loosening in cementless THA? Clin Orthop Relat Res 2018; 476: 1212–1220. 29481346 10.1007/s11999.0000000000000208 PMC6263580

[bibr6-11207000241239914] Lindgren V Garellick G Kärrholm J , et al. The type of surgical approach influences the risk of revision in total hip arthroplasty: a study from the Swedish Hip Arthroplasty Register of 90,662 total hip replacements with 3 different cemented prostheses. Acta Orthop 2012; 83: 559–565. 23116440 10.3109/17453674.2012.742394 PMC3555460

[7] Meneghini RM Elston AS Chen AF , et al. Direct anterior approach: risk factor for early femoral failure of cementless total hip arthroplasty: a multicenter study. J Bone Joint Surg Am 2017; 99: 99–105. 28099299 10.2106/JBJS.16.00060

[8] Abe H Sakai T Takao M , et al. Difference in stem alignment between the direct anterior approach and the posterolateral approach in total hip arthroplasty. J Arthroplasty 2015; 30: 1761–1766. 25956522 10.1016/j.arth.2015.04.026

[bibr9-11207000241239914] Cheng TE Wallis JA Taylor NF , et al. A prospective randomized clinical trial in total hip arthroplasty—comparing early results between the direct anterior approach and the posterior approach. J Arthroplasty 2017; 32: 883–890. 27687805 10.1016/j.arth.2016.08.027

[10] Hoskins WT Bingham RJ Lorimer M , et al. The effect of size for a hydroxyapatite-coated cementless implant on component revision in total hip arthroplasty: an analysis of 41,265 stems. J Arthroplasty 2020; 35: 1074–1078. 31787355 10.1016/j.arth.2019.10.060

[11] Dietrich M Kabelitz M Dora C , et al. Perioperative fractures in cementless total hip arthroplasty using the direct anterior minimally invasive approach: reduced risk with short stems. J Arthroplasty 2018; 33: 548–554. 28993084 10.1016/j.arth.2017.09.015

[12] Tamaki T Jonishi K Miura Y , et al. Cementless tapered-wedge stem length affects the risk of periprosthetic femoral fractures in direct anterior total hip arthroplasty. J Arthroplasty 2018; 33: 805–809. 29107490 10.1016/j.arth.2017.09.065

[13] Thien TM Chatziagorou G Garellick G , et al. Periprosthetic femoral fracture within two years after total hip replacement: analysis of 437,629 operations in the nordic arthroplasty register association database. J Bone Joint Surg Am 2014; 96: e167. 10.2106/JBJS.M.00643 25274795

[14] Jämsen E Eskelinen A Peltola M , et al. High early failure rate after cementless hip replacement in the octogenarian. Clin Orthop Relat Res 2014; 472: 2779–2789. 24771260 10.1007/s11999-014-3641-7 PMC4117887

[15] Tanzer M Graves SE Peng A , et al. Is cemented or cementless femoral stem fixation more durable in patients older than 75 years of age? A comparison of the best-performing stems. Clin Orthop Relat Res 2018; 476: 1428–1437. 29683803 10.1097/01.blo.0000533621.57561.a4 PMC6437589

[16] Clauss M Breusch S . The ‘French paradox’ may not be a paradox after all–but for what reason? Bone Joint Res 2019; 8: 1–2. 30800293 10.1302/2046-3758.81.BJR-2018-0235 PMC6359881

[bibr17-11207000241239914] Harvey E Tanzer M Bobyn J . Femoral cement grading in total hip arthroplasty. J Arthroplasty 1998; 13: 396–401. 9645519 10.1016/s0883-5403(98)90004-3

[bibr18-11207000241239914] Malchau H Herberts P Eisler T , et al. The Swedish total hip replacement register. J Bone Joint Surg Am 2002; 84-A(Suppl. 2): 2–20. 12479335 10.2106/00004623-200200002-00002

[bibr19-11207000241239914] Sanghrajka AP Whittingham-Jones P Higgs D , et al. Anterior or posterior: does the surgical approach to the hip influence the quality of the femoral cement mantle? Hip Int 2006; 16: 67–74. 19219782 10.5301/hip.2008.3541

[20] Schuroff AA Deeke M Pedroni MA , et al. Radiographic evaluation of cementation technique using polished, conical, triple-tapered femoral stem in hip arthroplasty. Rev Bras Ortop 2017; 52(Suppl. 1): 40–45. 10.1016/j.rboe.2017.08.019 PMC5620013 28971085

[21] Hoskins W van Bavel D Lorimer M , et al. Polished cemented femoral stems have a lower rate of revision than matt finished cemented stems in total hip arthroplasty: an analysis of 96,315 cemented femoral stems. J Arthroplasty 2018; 33: 1472–1476. 29310918 10.1016/j.arth.2017.12.002

[22] Zijlstra WP De Hartog B Van Steenbergen LN , et al. Effect of femoral head size and surgical approach on risk of revision for dislocation after total hip arthroplasty: an analysis of 166,231 procedures in the Dutch Arthroplasty Register (LROI). Acta Orthop 2017; 88: 395–401. 28440704 10.1080/17453674.2017.1317515 PMC5499330

[23] Ennin KA Elsharkawy KA Dasgupta S , et al. Cemented femoral stem fixation through the anterior approach has fewer early complications than cementless fixation. Bone Joint J 2021; 103-B(Suppl. B): 33–37. 34192902 10.1302/0301-620X.103B7.BJJ-2020-2230.R1

[24] McGoldrick NP Fischman D Nicol GM , et al. Cementing a collarless polished tapered femoral stem through the anterior approach: evaluation of cement mantle quality and component alignment. Bone Joint J 2021; 103-B(Suppl. B): 46–52. 10.1302/0301-620X.103B7.BJJ-2020-2394.R1 34192917

[25] Bartlett GE Gill HS Murray DW , et al. In vitro influence of stem surface finish and mantle conformity on pressure generation in cemented hip arthroplasty. Acta Orthop 2009; 80: 139–143. 19404792 10.3109/17453670902947382 PMC2823161

[26] Aspenberg P van der Vis H . Fluid pressure may cause periprosthetic osteolysis: particles are not the only thing. Acta Orthop Scand 1998; 69: 1–4. 9524506 10.3109/17453679809002344

[27] Athanasou NA . The pathobiology and pathology of aseptic implant failure. Bone Joint Res 2016; 5: 162–168. 27146314 10.1302/2046-3758.55.BJR-2016-0086 PMC4921050

[28] Melman WP Mollen BP Kollen BJ , et al. First experiences with the direct anterior approach in lateral decubitus position: learning curve and 1 year complication rate. Hip Int 2015; 25: 251–257. 25684251 10.5301/hipint.5000221

[bibr29-11207000241239914] den Hartog YM Vehmeijer S . High complication rate in the early experience of minimally invasive total hip arthroplasty by the direct anterior approach. Acta Orthop 2013; 84: 116–117. 10.3109/17453674.2013.773412 PMC3584595 23409847

[30] Panichkul P Parks NL Ho H , et al. New approach and stem increased femoral revision rate in total hip arthroplasty. Orthopedics 2016; 39: e86–e92. 26726989 10.3928/01477447-20151222-06

[31] Junnila M Laaksonen I Eskelinen A , et al. Implant survival of the most common cemented total hip devices from the Nordic Arthroplasty Register Association database. Acta Orthop 2016; 87: 546–553. 27550058 10.1080/17453674.2016.1222804 PMC5119435

[32] Schmitz M Bronsema E de Kam D , et al. Results of the cemented Exeter femoral component in patients under the age of 40: an update at ten to 20 years’ follow-up. Bone Joint J 2017; 99-B: 192–198. 28148660 10.1302/0301-620X.99B2.38045

[33] Kenanidis E Kaila R Poultsides L , et al. Quality of the femoral cement mantle in total hip arthroplasty using the direct anterior hip approach. Arthroplast Today 2020; 6: 601–606.e2. 10.1016/j.artd.2020.02.012 PMC7502567 32995408

[34] Mayr E Krismer M Ertl M , et al. Uncompromised quality of the cement mantle in Exeter femoral components implanted through a minimally-invasive direct anterior approach: a prospective, randomised cadaver study. J Bone Joint Surg Br 2006; 88: 1252–1256. 16943482 10.1302/0301-620X.88B9.17538

[35] Brun OL Sund HN Nordsletten L , et al. Component placement in direct lateral vs minimally invasive anterior approach in total hip arthroplasty: radiographic outcomes from a prospective randomized controlled trial. J Arthroplasty 2019; 34: 1718–1722. 31053468 10.1016/j.arth.2019.04.003

[36] Sevaldsen K Husby OS Lian ØB , et al. Is the French Paradox cementing philosophy superior to the standard cementing? A randomized controlled radiostereometric trial and comparative analysis. Bone Joint J 2022; 104-B: 19–26. 34969272 10.1302/0301-620X.104B1.BJJ-2021-0325.R2 PMC8779947

[37] Sevaldsen K S Husby O B Lian Ø , et al. Does the line-to-line cementing technique of the femoral stem create an adequate cement mantle? Hip Int 2021; 31: 618–623. 32551930 10.1177/1120700020934368 PMC8488641

[38] Tay K Tang A Fary C , et al. The effect of surgical approach on early complications of total hip arthoplasty. Arthroplasty 2019; 1: 5. 35240769 10.1186/s42836-019-0008-2 PMC8787926

[39] Wilson EJ Fricka KB Ho H , et al. Early practice all-cause complications for fellowship-trained anterior hip surgeons are not increased when compared to “gold standard” experienced posterior approach surgeons. J Arthroplasty 2023; 38: 2355–2360. 37179026 10.1016/j.arth.2023.05.008

[40] Hoskins W Bingham R Vince K . A systematic review of data collection by national joint replacement registries: what opportunities exist for enhanced data collection and analysis? JBJS Rev 2023; 11: e23.00062. 10.2106/JBJS.RVW.23.00062 37956205

